# Antibiofilm Power of Basil Essential Oil Against Fish-Originated Multidrug-Resistant *Salmonella* and *Bacillus* spp.: Targeting Biofilms on Food Contact Surfaces

**DOI:** 10.3390/foods14101830

**Published:** 2025-05-21

**Authors:** Valentina Pavone, Francisco Emilio Argote-Vega, Waleed Butt, Junior Bernardo Molina-Hernandez, Domenico Paludi, Johannes Delgado-Ospina, Luca Valbonetti, José Ángel Pérez-Álvarez, Clemencia Chaves-López

**Affiliations:** 1 Deparment of Bioscience and Technology for Food, Agriculture and Environment, University of Teramo, Via R. Balzarini 1, 64100 Teramo, Italy; vpavone@unite.it (V.P.); buttwaleed177@gmail.com (W.B.); lvalbonetti@unite.it (L.V.); 2 IPOA Research Group, Centro de investigación e Innovación Agroalimentaria y Agroambiental de la UMH (CIAGRO), Miguel Hernández University, 03202 Orihuela, Alicante, Spain; argote_71@hotmail.com (F.E.A.-V.); ja.perez@goumh.umh.es (J.Á.P.-Á.); 3 Department of Agricultural and Food Sciences, University of Bologna, 47521 Cesena, Italy; 4 Department of Veterinary Medicine, University of Teramo, 64100 Teramo, Italy; dpaludi@unite.it; 5 Grupo de Investigación Biotecnología, Facultad de Ingeniería, Universidad de San Buenaventura Cali, Carrera 122 # 6-65, Cali 76001, Colombia; jdelgado1@usbcali.edu.co

**Keywords:** linalool, rainbow trout, fish-based model system, biofilm inhibition, pathogenic and spoilage bacteria, biofilm morphotype, food contamination

## Abstract

The antimicrobial and antibiofilm efficacy of two *Ocimum basilicum* L., essential oils sourced from Colombia (BEOC) and Italy (BEOI), was evaluated against multidrug-resistant fish isolates of *Salmonella enterica* subsp. *salamae*, *Bacillus thuringiensis*, and *Bacillus oceanisediminis*—species for which such activity has not been previously reported. Using a fish-based model system (FBMS), we found that BEOI, rich in linalool (69.86%), exhibited stronger antimicrobial activity than camphor-dominated BEOC (24.61%). The antimicrobial effects of both EOs were strain- and concentration-dependent, with minimum bactericidal concentration (MBC) 3.75–15.0 µL/mL for BEOI and 15.0–30.0 µL/mL for BEOC. Pure linalool showed even greater potency (MBC: 0.0125 to 0.025 µL/mL). Confocal laser scanning microscopy revealed that BEOI induced severe membrane damage (27% of the cells within 1 h), ultimately leading to the death of 96% of the cells after 24 h. Biofilm formation, assessed in both FBMS and tryptone soy broth (TSB), was strain-dependent, with FBMS promoting higher biofilm production than TSB. Moreover, significant differences in biofilm morphotypes were observed, with the morphotype PDAR (pink dry and rough), characterized by only cellulose, being the most frequently exhibited by the strains (7/15), while BDAR (brown dry and rough), characterized by only curli, was the least expressed (7/15); the remaining strains presented morphotype RDAR. In addition, the strains in polystyrene surfaces accumulated more biomass than stainless steel 304. Notably, BEOI and linaool significantly reduced biofilm formation across all strains, with a reduction of 90% in *S. enterica* subsp. *salamae* strains (TJC19 and TJC21. These strains with the RDAR phenotype likely contribute to their strong biofilm-forming capacity. Our findings highlight BEOI’s potential as a natural anti-biofilm agent in food processing environments, offering a promising strategy to combat multidrug-resistant bacteria biofilm-related challenges in the food industry.

## 1. Introduction

The fishing industry is facing major challenges due to the formation of biofilms by pathogenic and spoilage bacteria, which can lead to decomposition processes and diseases that affect both fish and consumers. A biofilm is a structured and complex community of bacteria and other microorganisms embedded in an organic matrix and adhering to a surface. These microorganisms appear to benefit greatly from surface association, which is an ancient, ubiquitous, and fundamental survival mechanism that enhances access to food sources, promotes interaction with other organisms, and stabilizes the environment [1]. Different types of surfaces can be colonized by a variety of microorganisms, which can lead to the formation of biofilms and the development of specialized processes within them. It is well established that microorganisms gain advantages from such a unique lifestyle, including resistance to desiccation, improved antibiotic resistance, higher nutrient concentration, and defense against predators [2]. Furthermore, the benefits of cell–cell contact may be the reason for the shared lifestyle of the surface association [3].

Biofilms are not limited to natural ecosystems. Rather, food processing environments provide an optimal milieu for biofilm development, as these facilities include surfaces routinely or continuously inundated with nutrient-rich liquids. In fact, modern food processing facilities serve as conducive habitats for the establishment of biofilms on food contact surfaces. This is mainly due to the complicated architecture of the production facilities, the long production time, the high product output, and the large surface areas that favor the spread of biofilms [2]. In this context, bacteria associated with biofilms on food processing surfaces and utensils such as pipework, tables, philters, conveyor belts, and knives may exhibit resistance to various physical interventions such as desiccation and irradiation, mechanical methods such as cleaning protocols, and chemical treatments including sanitizers and disinfectants [4]. Research shows that microorganisms on equipment and utensils in food processing facilities are due to inadequate hygiene practices, which hurt public health and the economy [4,5].

Given concerns about the safety of consuming fish and fish products that are colonized, the study of biofilm formation in these organisms has attracted increasing scientific interest [6]. It is well known that biofilms increase the pathogenicity and resilience of bacteria; a variety of strategies have been developed to address this challenge. While prevention is considered the most effective method to control biofilm formation, it is equally important to research and develop methods to eradicate existing biofilms. The predominant approach in the industry involves a synergistic application of chemical and physical strategies. The physical techniques include brushing, scrubbing, foaming, water jets, pulsed electric fields, another others [7]. Common chemical agents include hydrogen peroxide, peracetic acid, quaternary ammonium compounds, and chlorine-based disinfectants [8]. In recent years, the use of natural and environmentally friendly alternatives such as enzymes, bacteriophages, bacteriocins, quorum sensing inhibitors, and essential oils has been proposed [9,10,11].

The food industry is particularly concerned about the ability of biofilms to provide bacterial cells with resistance against antimicrobial agents [12]. As a result, regular cleaning and disinfection procedures are frequently insufficient in combating the bacteria that create biofilms. Since synthetic antimicrobials are typically discovered to be hazardous and unfriendly to the environment, comprehensive searches from natural materials are undertaken to discover effective antibacterial agents. In the current literature, the scientific focus on the anti-biofilm effect of essential oils has increased significantly, which is due to their diverse chemical components that can be used as antimicrobial agents not only against planktonic microorganisms but also against their sessile counterparts. In fact, essential oils can disrupt the biochemical metabolic pathways involved in biofilm formation, thus facilitating the containment and regulation of this phenomenon [4].

Our investigation focuses on essential oils from *Ocimum basilicum* used to reduce bacterial proliferation and biofilm development on two different surfaces commonly encountered in the food industry, specifically polystyrene (PS) and stainless steel (SS), using a fish-based model system (FBMS). Although a considerable number of studies on the inhibition of biofilm formation have focused primarily on well-documented pathogenic bacteria such as *Listeria monocytogenes, Staphylococcus aureus, Escherichia coli, Pseudomonas aeruginosa, Salmonella typhimurium,* and *Vibrio parahaemolyticus*, there is a conspicuous absence of research on *Salmonella enterica* subsp. *salamae* and *Bacillus* species, which are considered to be the most prevalent bacteria capable of adhering to surfaces and exhibiting a marked propensity for biofilm formation in various areas such as the food industry, medical practice, and aquatic systems [13,14], and which are implicated in food spoilage. Considering that pathogenic and spoilage microorganisms can be introduced into fish and fish products throughout the production and supply chain, mainly through the natural formation of biofilms, this study investigated the potential of *Ocimum basilicum* L. essential oil (BEO) to inhibit bacterial growth and its efficacy in suppressing biofilm production at a temperature of 15 °C simulating fish processing conditions, focusing on 15 different strains of *Salmonella enterica* subsp. *salamae*, *Bacillus thuringensis,* and *Bacillus oceanisediminis*, isolated from Rainbow trout (*Oncorhynchus mykiss*).

## 2. Materials and Methods

### 2.1. Microorganisms

#### 2.1.1. Isolation and Identification of Bacterial Strains

The bacterial strains were isolated from fresh rainbow trout (*Oncorhynchus mykiss*) from Laguna de la Cocha (Pasto, Colombia). The strains were isolated from the skin surface in aseptic conditions. The strain cultures were grown on Tryptic Soy Broth (TSB, Oxoid, Milan, Italy) at 20 °C and stored at −80 °C in cryogenic vats with glycerol 20% *v*/*v* (Sigma-Aldrich, St. Louis, MO, USA).

For the identification, the strains were grown in Brain Heart Infusion Broth (BHI) medium culture plates and incubated at 28 °C for 24 h. Genomic DNA was extracted using the DNA Extraction Kit (Euro Clone, ZYD6007 Zymo, Pero (MI), Italy). Molecular identification was performed by amplification of the 16S rRNA gene (600 bp) with the universal primer pairs (F) 5′-AGTTTGATCCTGGCTCAG-3′ and (R) 5′-ATTACCGCGGCTGCTGG-3′. The PCR reaction was performed in a total volume of 50 µL of a solution containing 0.25 µL 1X Wonder Taq Hot Start Reaction buffer (Euroclone), 1 µL 100µM Primer Forward, 1 µL 100 µM Primer Reverse, 10 µL DNA extract, and PCR water brought to a final volume. Amplification was carried out in a thermal cycler (Bio-Rad) as follows: 2 min at 95 °C, 25 cycles of: 1 min at 95 °C, 1 min at 55 °C, 1 min at 72 °C, and a final extension step of 7 min at 72 °C. Finally, the sequences obtained were aligned to other closely related bacterial species deposited in the NCBI database using the BlastN program. All primers used were purchased from Sigma Aldrich (Saint Louis, MO, USA).

#### 2.1.2. Antibiotic Resistance of the Strains

From the pure and fresh growth of the strains, 0.5 McFarland suspensions were prepared with sterile saline solution (1 × 10^8^ to 2 × 10^8^ CFU/mL). The suspension was then inoculated onto Müeller–Hinton agar for modified Kirby–Bauer disk diffusion analysis according to the Clinical and Laboratory Standards Institute (CLSI) guidelines [15] and using the VITEK^®^ 2 automated system (BioMèrieux, Marcy L’Etoile, France) according to the manufacturer’s instructions. The antibiotics tested were: Erythromicin (E), Amoxicillin (AML), Cefuroxime (CXM), Tetracycline (TE), Teicoplanin (TEC), Chloramphenicol (C), Kanamicyn (K), Doxycyclin (DXT), Oxacillin (OX), Gentamicin (CN), Linezolid (LNZ), Enrofloxacin (ENR), Nalidixic acid (Na), Clindamycin (CD), Rifampicin (RD), Amikacin (AK), Cephalothin (KF), Ciprofloxacin (CIP), Sulphamethoxalone (SXT), Bacitracin (B), Flumequine (UB), Spiramycin (SP), Oleandomycin (OL), Fusidic acid (FD).

### 2.2. Origin and Composition of O. basilicum Essential Oils

*Ocimum basilicum* L. essential oil from Colombia (BEOC) was provided by the University of San Buenaventura, Cali-Colombia, and was compared with the antimicrobial and antibiofilm activity of *O. basilicum* essential oil from Italy (BEOI) purchased at a local market in Teramo city.

The composition of the essential oils was determined by gas chromatography coupled to mass spectrometry (GC-MS) with an AT6890 plus series gas chromatograph (Agilent Hewlett-Packard Technologies, Palo Alto, CA, USA) and a mass selective detector (Agilent Technologies, MSD 5970, Hewlett-Packard, Geneva, Switzerland) using solid phase microextraction (SPME) following the method previously described by Chaves-López et al. [16]. The SPME fiber (75 mm, carboxen/polydimethylsiloxane, CAR/PDMS) was conditioned for 30 min at 270 °C onefold and, for 15 min at 270 °C for post-conditioning. The fiber was exposed to the vial with headspace for 40 min at 50 °C before injecting into the GC. The injector and FID temperatures were at 250 °C, and the detector temperature was 220 °C. Helium was used as a carrier gas at a flow rate of 1 mL min 1. The GC oven temperature program was 40 °C for 2 min, ramped to 200 °C at 10 °C/min, then ramped from 200 to 250 °C at 15 °C per min, and finally held at 250 °C for 5 min. The NIST software was used in the identification process, matching mass spectra of components. For the calculation of relative amounts of components, integration of the GC peak area was used with a correction factor.

### 2.3. Evaluation of the Antibacterial Activity

#### 2.3.1. Preparation of Fish-Based Model System (FBMS)

As it is essential to use representative food model media to identify potential interactions between essential oils (EOs) and food constituents that may influence their antimicrobial efficacy, a model system with fresh fish was used in this study, following the methodology described by Pilevar et al. [17]. Briefly, fresh trout filets were sourced from a local retailer, deboned, cut into smaller portions, and then boiled with distilled water at a ratio of 1:2 (*w*/*v*) for 20 min. The resulting suspension was then filtered and buffered with 9.75 g/L dibasic potassium phosphate (K_2_HPO_4_) and 5.98 g/L monobasic potassium phosphate (KH_2_PO_4_). The pH of the broth was adjusted to 6.3 using hydrochloric acid (HCl). Finally, the broth was sterilized at 121 °C for 15 min.

The composition of the principal components of the media was analyzed as follows: The ninhydrin method described by Moore and Stein [18] was used to determine the free amino acids, and the BCA Protein Assay (MERK, Darmstadt, Germany) was used for the quantitation of total protein. The soluble peptide content was measured using the Peptide Assay Kit (MoBiTec GmbH, Goettingen, Germany). Lipids were determined with the method proposed by Cheng et al. [19] and Ash in a muffle at 600 °C for 3 h.

#### 2.3.2. Determination of Minimum Inhibitory Concentration (MIC)

The essential oils and linalool were dissolved in sterile PBS plus Tween 80 (10% of the oil concentration), to obtain a concentration of 50 mg/g, from which serial dilutions were made in sterile PBS. Since linalool was the most abundant component of the BEOI, the chemotype that showed major antimicrobial activity, we also tested this compound alone.

The Minimum Inhibitory Concentration (MIC) of the essential oils (EOs) and linalool after 168 h at 15 °C was determined by the microdilution method [20], using a 96-well microtiter plate (Corning Incorporated, Kennebunk, ME, USA). In each well of the polystyrene microtiter plate, an aliquot of 100 μL of FBMS, added with 0.01% of the redox indicator 2,3,5-triphenyltetrazolium chloride (TTC, 0.01%, Sigma-Aldrich, Milan, Italy), was introduced. Subsequently, 100 μL of the stock essential oil (EO) solution or linalool was dispensed into the initial well, and serial dilutions were conducted to realize a concentration range of 40–1.25 μL/mL for the essential oils and 2.0–0.0005 μL/mL. Following this, a bacterial suspension of 100 μL was incorporated, reaching a total volume of 200 μL within each well. For each strain, a positive control (comprising 100 μL of medium in conjunction with 100 μL of inoculum) and a negative control (consisting of 200 μL of medium) were established. The MIC value was considered as the lowest concentration at which no microbial growth was observed, as a consequence of TTC (2,3,5-triphenyltetrazolium chloride, Sigma-Aldrich, Milan, Italy) red discoloration, previously added in a ratio of 1 μL/mL of solution 1% of TTC. The experiments were replicated three times. The most effective EOs, based on MIC results, were selected for further studies.

From the determination of the Minimal Bactericidal Concentration (MBC), a volume of 0.1 mL was removed from the wells in the microtiter plates where no growth was observed after 168 h at 15 °C and inoculated onto the surface of TSA plates (Oxoid). They were incubated for 48 h at 37 °C, with MBC being taken to be the lowest concentration of the substance at which no colonies formed under these conditions. Three replicates were performed for each strain and antimicrobial compound. In order to compare the efficacy of BEO with a commercial sanitizer, we used sodium hypochlorite at a concentration of 10 µL/mL

#### 2.3.3. Confocal Laser Scanning Microscopy (CLSM) Analysis

To clarify and quantify the influence of BEOI, we selected a single strain of each species analyzed, such as *B. oceanisediminis* TJC18, *B. thuringensis* TJC2, and *S. enterica* subsp. *salamae* TJC19, which were subjected to MIC values. Confocal laser scanning microscopy was used to count the live and dead cells after the BEOI treatments by using propidium iodide (PI) and carboxyfluorescein diacetate (CFDA), as reported by Molina-Hernandez et al. [21].

Bacterial cells were standardized (approximately 1 × 10^6^ CFU/mL), inoculated in FBMS, and exposed to BEO at MIC values for 1 h and 24 h. Then, samples were stained with CFDA and PI. Finally, 10 μL of bacteria were deposited in polyresin-covered slides and observed with the Nikon A1R confocal imaging system (Nikon Corp., Tokyo, Japan) as reported previously. Isopropanol at 70% *v*/*v* for 1 h was used as a positive control as suggested by Lee et al. [22].

### 2.4. Biofilm Bioproduction

#### 2.4.1. Biofilm Morphotype

To determine the biofilm morphotype of the different strains, the method described by Choong et al. [23] was performed using Congo Red agar (CR-agar), which was prepared by supplementing LB agar w/o salt with CR and Coomassie brilliant blue G-250. Biofilm formation was initiated by positioning 10 μL aliquots from liquid, stationary-phase cultures onto CR-agar in the Petri dish. After incubation at 15 °C for 7 days, morphotypes based on curli and cellulose expression were determined by visual inspection and documented by photography. In addition to determining the production of cellulose, we perform the Calcofluor assay, using LB agar w/o salt supplemented with 2 μL Calcofluor White stain (Calcofluor White M2R (1 g/L), Evans’s blue (0.5 g/L), Sigma-Aldrich, Stockholm, Sweden) per mL agar was added.

#### 2.4.2. Comparative Biofilm Production on Polystyrene Surface Using TSB and FBMS

The biofilm formation was investigated using the two synthetic media, TSB and FBMS. Thus, 200 µL of each strain was grown singularly in TSB or FBMS for 144 h at 15 °C. Inoculum was added into each well (96-well polystyrene microplates) using TSB and FBMS as a culture medium. After the incubation time, the planktonic cells were removed from each sample, and then each well was washed with 10 mM PBS pH 7.4. Total biomass was quantified by crystal violet assay at OD_590_, as reported by Rossi et al. [24]. The experiment was performed in five replicates.

#### 2.4.3. Biofilm Formation on Stainless Steel (SS)

To detect the biofilm formation on SS, we used coupons of 2 × 2 × 0.1 (AISI 316, the second most commonly available and widely used stainless steel type), previously cleaned according to the procedure described by Maggio et al. [3]. Briefly, coupons were degreased with acetone, etched in 5 N HCl (15 min), washed with detergent, rinsed with deionized water, air-dried, and autoclaved (121 °C, 15 min). Each coupon was placed in a sterile glass vial containing 5 mL of cheese medium inoculated with each culture (negative controls: non-inoculated media). The samples were incubated at 15 °C for 144 h (n = 3 per time point: 0, 48, 72, 96, and 168 h). Also, in this case, the biofilm formation was evaluated after 144 h at 15 °C.

#### 2.4.4. Efficacy of BEOI and Linalool on the Biofilm Inhibition

To evaluate the effectiveness of BEOI and linalool on biofilm formation on SS, coupons (2 × 2 × 0.1 cm^3^) were used. Each coupon was individually introduced into a sterile glass Petri dish with 5 mL of FBMS and inoculated with the corresponding strain. Also, negative control samples with non-inoculated FBMS were included. The samples were then incubated at 15 °C for 144 h. Measurement of the biofilm formation was performed as described by Rossi et al. [25].

### 2.5. Effect of Basil Essential Oil and Linalool on Biofilm Production

For the evaluation of the effect of BEOI and linalool on the biofilm formation using FBMS, 200 µL of the different strains grown in FBMS were singularly added into each well of 96-well polystyrene microplates and SS coupons using the fish-based model system (FBMS) as a culture medium. The bacterial suspensions were added with 10 μL/mL of BEOI at MIC, respectively, and incubated at 15 °C for 144 h. The control samples contained untreated inocula in the FBMS. At the end of incubation, the planktonic cells were removed, and 100 μL of the suspension was taken to perform serial dilutions that were distributed on FBMS and inoculated at 15 °C for 96 h. Then, each well of the microplates was washed with 10 mM PBS pH 7.4. Total biofilm biomass was quantified by crystal violet assay at OD_590_, as reported above. The experiment was performed in five replicates.

### 2.6. Data Analysis

All data were processed using XLSTAT, a Microsoft Office Excel add-in software (XLSTAT 2021, Addinsoft, Paris, France). The differences between biofilm production of the different strain in two different media (FBMS and TSB) as well as the differences between the biofilm formation in two different surfaces (PL and SS) were determined by a one-way analysis of variance (ANOVA), followed by Tukey post hoc test (HSD) that was performed to analyze the statistical difference among means.

A three-way ANOVA analysis using XLSTAT 2021 (Addinsoft, Paris, France) was selected to investigate factor effects (strain, treatment, surface) and interactions among them. In all cases, the threshold for significance was 5%. Post hoc comparisons were performed using the Tukey post hoc test (HSD).

## 3. Results

### 3.1. Microorganisms

#### 3.1.1. Bacterial Identification Using 16S rDNA Gene Sequences

The bacterial strains used in this study were assigned to *Bacillus oceanisediminis* (5 strains), *Bacillus thuringiensis* (5 strains), and *Salmonella enterica* subsp. *salamae* (5 strains). The identification of bacterial isolates with 99% and 100% homology is shown in Table S1. It is generally recognized that *Salmonella* spp. is the most common pathogen in fish, followed by *L. monocytogenes, Vibrio* spp., *Yersinia* spp., *C. botulinum*, *S. aureus,* and *Aeromonas* spp., which are the primary bacterial etiology for fish-related outbreaks [6]. In particular, *S. enterica* subsp. *salamae* is considered an opportunistic pathogen [26], which has been isolated from aquatic environments and fish and has shown antibiotic resistance to a range of antimicrobial agents [27]. This species has a lower virulence compared to the important virulence serotypes, such as *S. typhimurium* and *S. enteritidis*. It should be emphasized that Salmonella infections represent a serious global health and economic burden, with approximately 95 million annual cases worldwide, including life-threatening bacteremia (3–8% of infections) and increasing antibiotic resistance. In the EU, it is the second most common zoonosis (after campylobacteriosis), causing over 91,000 reported cases annually and €3 billion in annual healthcare costs, productivity losses, and outbreak management [28]. These impacts place a burden on public health systems and the food industry and emphasize the urgency of improved prevention strategies.

On the other hand, species of the genus *Bacillus* have already been identified in fish [13], and their prevalence can be attributed to their ubiquitous distribution and ability to form endospores, which facilitates their survival in both fish and fish processing [29]. *B. oceanisediminis* has already been isolated from marine sediment samples in the South China Sea [30], while *B. thuringiensis* is considered one of the most widespread opportunistic pathogens of *Bacillus* species infecting both humans and animals; it has been isolated from freshwater fish [13,31]. In addition, this species produces proteins that are toxic and have a high specificity towards various pests, including protozoa, insects, helminths, and mites, which are important for both agriculture and veterinary medicine [32].

#### 3.1.2. Antibiotic Resistance of *B. thuringensis*, *B. oceanisediminis*, and *S. enterica* subsp. *salamae*, Strains

**Figure 1:**
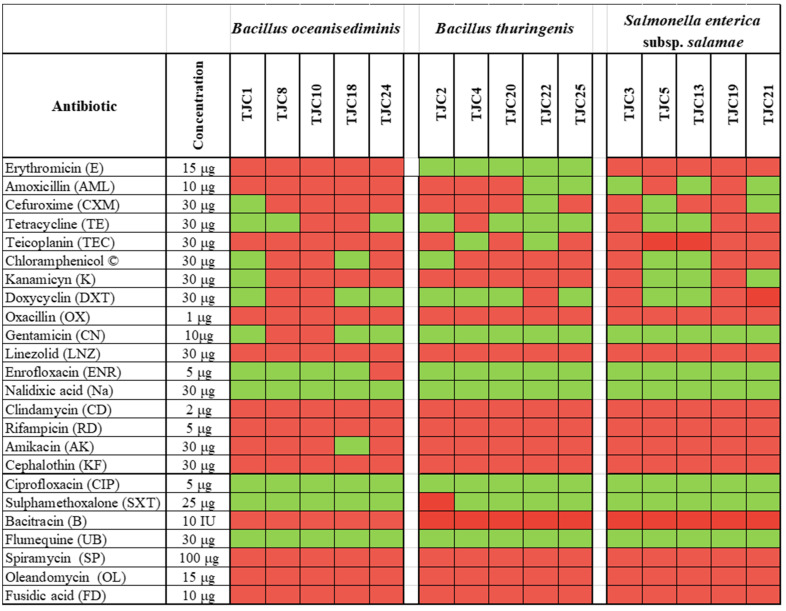
Antibiotic resistance of *B. oceanisediminis*, *B. thuringiensis*, and *Salmonella enterica* subsp. *salamae* strains. Red color: resistant strain; green color: sensible strain.

Freshwater ecosystems are susceptible to contamination by antibiotic residues originating from various sources, e.g., agricultural runoff, sewage discharge, and leaching from adjacent farmland [33]. Consequently, the freshwater environment can turn into a reservoir where antibiotics exert selective pressure on microorganisms and thus pose a significant risk to public health. Given this concern, we investigated the antibiotic resistance profiles of the bacterial strains used in this study. As shown in Figure 1, a significant proportion of the strains exhibited resistance to multiple antibiotics. Antibiogram analysis revealed that all strains of *B. oceanisediminis* and *S. enterica* subsp. *salamae*, but none of the five strains of *B. thuringensis* strains, were resistant to erythromycin. In addition, the tested strains exhibited pronounced “multiple antibiotic resistance” (MAR); in particular, the MAR of *Salmonella enterica* subsp. *salamae* was characterized by resistance to E, TEC, OX, LNZ, CD, RD, KF, B, SP, OL, and FD, while the resistance profile for *B. thuringensis* included K, DX, LNZ, CD, RD, KF, B, SP, OL, and FD. In addition, the strains of *B. oceanisediminis* showed MAR to E, AML, TEC, OX, LNZ, CD, RD, KF, B, SP, OL, and FD. Notably, despite resistance to multiple antibiotics of different classes, all strains remained susceptible to fluoroquinolones (Ciprofloxacin, Enrofloxacin), sulfonamides (Sulfamethoxalone), and quinolones (Nalidixic acid, Flumequine). These findings align with prior reports of MDR prevalence in aquatic environments. Lakes, in particular, are known to have the ability to accumulate antibiotic resistance genes (ARGs) to a greater extent than rivers due to their longer water residence times, which prolong the persistence of pollutants (independent of vertical transport mechanisms such as sedimentation rates) [34].

### 3.2. Essential Oils

#### 3.2.1. Chemical Composition of the *O. basilicum* Essential Oils

The essential oils of basil (BEOs) were analyzed using gas chromatography-mass spectrometry (GC-MS). Table 1 contains a comprehensive list of the essential oil components with the retention indices (RIs). The results show that the predominant compounds in BEOC consist of camphor (24.61%), followed by eucalyptol (24.12%), ρ-allylanisole (10.92%), and α-bisabolene (5.59%). Conversely, the composition of BEOI revealed linalool (69.86%) as the main component, followed by 1,8-cineole (12.91%) and estragole (7.89%). Other compounds such as τ-cadinol, terpinolene, 4-terpineol, iso-bornyl acetate, and coumarin were detected in relatively small quantities.

It is well known that the chemical composition of essential oils now varies significantly due to multiple environmental factors, including microclimatic conditions, geographical distribution, altitudinal or longitudinal gradients, and environmental stress factors such as increased soil salinity and drought, as well as changes in average annual temperatures. These variations pose significant challenges for comparative analyses of essential oil properties. In this context, Basil essential oil (BEO) exhibits significant chemical variability that can be influenced by geographical, climatic, and cultivation factors. Research indicates distinct chemotypes—such as methyl chavicol/eugenol-dominated versus linalool-rich varieties—vary across regions, with tropical climates favoring higher eugenol content and temperate zones promoting linalool dominance [35]. Altitude and UV exposure further alter EO composition [36], while soil type plays a decisive role. For instance, A. Tursun [37] reported that methyl cinnamate concentrations differed significantly between soil types, peaking at 46.03% in loamy sandy soils compared to 42.33% in sandy-clay loam. Additionally, nitrogen fertilization impacts BEO profiles: linalool content ranged from 58% (100 kg N/ha) to 61% (150 kg N/ha), whereas naphthalene levels peaked at 13.87% (50 kg N/ha) before declining to 11.58% (150 kg N/ha) [38]. Given these fluctuations, studies assessing BEO bioactivity (e.g., antimicrobial efficacy) must account for chemotypes and environmental influences to ensure reproducible and generalizable conclusions. Another problem that frequently occurs in the basil research literature is the lack of standardization of extraction methods. For example, there is no consensus on the choice of a plant organ to be studied, appropriate drying techniques, homogenization procedures, and the methods used to identify the chemical constituents. This methodological heterogeneity hinders direct comparison between studies and underscores the need for established protocols in essential oil research.

Numerous published studies have indicated the presence of linalool as the predominant constituent in the essential oils of *O. basilicum*. In this context, a study by Cheliku et al. [39] found in five Basil cultivars (*Ocimum basilicum*) from Albania considerable amounts of linalool (26.17–50.16%), 1,8-cineole (1.25–13.00%), cis-thujone (0.25–6.56%), methyl chavicol (0.66–33.05%), eugenol (0.17–13.73%), amount others. Amor et al. [40] further demonstrated that the most abundant component in basil oil is linalool (41.3%), followed by 1,8-cineole (9.6%), (Z)-isoeugenol (5.9%), 1-epi-cubenol (4.8%), α-transbergamotene (4.6%), and (Z)-anethole (3.2%). The same authors also demonstrated that basil has pronounced antimicrobial properties, which could be due to the high content of linalool, both in its unencapsulated form and in microencapsulated form. Massoud et al. [41] reported that the main constituent of the essential oil of French basil is linalool (11–28%), followed by eucalyptol (8–25%), α-terpinol (0.44–4.6%), and bergamotene (4.0–7.2%). In a study by Viña and Murillo [42], using 12 different varieties of basil essential oil from Colombia, it was reported that ten of these varieties were characterized by a significant presence of methyl cinnamate (35–80%), which delineated the chemotype for these varieties. Only one cultivar exhibited the caryophyllene chemotype, while another exhibited the linalool chemotype. Based on a comparative analysis with the existing literature, the BEOC chemotype appears to have a fundamentally different essential oil composition, which could be categorized as the camphor/eucalyptol chemotype according to Grayer’s chemotype classification system, while BEOI belongs to the linalool chemotype.

#### 3.2.2. Antimicrobial Activity of the Essential Oils

The evaluation of the BEOC and BEOI activity on bacterial growth in FBMS at 15 ± 1 °C (Table 2) revealed that BOEI exhibited greater efficacy, with MIC values ranging from 1.87 μL/mL to 15 μL/mL. In contrast, BEOC showed significantly lower antimicrobial activity (*p* < 0.05), with MIC values ranging from 15 to 30 μL/mL across the tested strains. Despite these differences, prolonged cultivation of up to 10 days confirmed that BEO remained active against all the tested bacteria as indicated by their MBC values.

The strong antimicrobial activity of BEO has previously been observed in Gram-positive and Gram-negative bacteria. However, this is the first study in which BEO is used against the species *B. oceanisediminis, B. thuringiensis*, and *S. enterica* subsp. *salamae*. Existing literature highlights the antibacterial activity of BEO, particularly the linalool chemotype. For example, Snoussi et al. [43] reported MIC values between 0.019 and 0.039 mg/mL against strains of *Vibrio* spp. in Mueller–Hinton broth 1% NaCl medium at 37 °C, though higher concentrations (>2.5 to >10 mg/mL) were required for bactericidal effect. Similarly, BEO was found to be highly effective against *E. coli, S. typhi, S. paratyphi, Proteus vulgaris*, and *S. aureus* [44], while inhibiting the growth of *S. enteritidis* at 20.0–80.0 μg/mL in BHI [45]. In contrast, higher MIC values (5.0 mg/mL) were observed for *Clostridium perfringens* in a reinforced clostridial medium [46], whereas Lv et al. [47] reported lower MIC values (1.25 μL/mL for BEO for *E. coli* and 0.625 μL/mL for *S. aureus* in nutrient agar. Seasonal variations also influence BEO’s antimicrobial activity. Hussain et al. [48] found that oils collected in autumn and winter exhibited stronger effects, which were correlated with higher linalool concentration (between 56.7% and 60.6%) compared to oils collected in summer and spring. Bassolé et al. [49] demonstrated that 2.1 mg/mL linalool and 6.7 mg/mL eugenol, key compounds in their BEO chemotype, had antimicrobial activity against *P. aeruginosa*. Given these findings, the superior efficacy of BEOI used in our study may stem from its high content of linalool (69.86%) and other oxygenated monoterpenes. Supporting this, it has been demonstrated that exposure of bacteria to linalool led to a disrupted membrane potential (MP), inducing leakage of alkaline phosphatase (AKP) in *S. putrefaciens* [50].

The biological activity of essential oils (EOs) is primarily attributed to phenolic compounds or their oxygen-substituted derivatives [51]. In this study, BEOI contained a high proportion of linalool (69.86 ± 1.89%), a monoterpene characterized by its low molecular weight and significant lipophilicity, indicating its potential to penetrate the cell membranes. Given these properties, we decided to evaluate the efficacy of linalool on bacterial growth and biofilm formation. As shown in Table 2, linalool had a strong effect across all the tested species, with MIC values ranging from 0.00625 to 0.025 μL/mL, though no significant differences were observed between species. This suggests that linalool effectiveness is also strain-dependent, consistent with prior findings. For instance, Gram-negative bacteria such as *Salmonella* and *E. coli* have demonstrated greater sensitivity to linalool than Gram-positive species such as *S. aureus* and *L. monocytogenes* [52], likely due to differences in their cell walls. It should be emphasized that they tested only one strain per species, as in most other studies, limiting broader generalizations. Mechanistically, Guo et al. [50] suggested that linalool inhibits key metabolic enzymes (e.g., G6PDH, MDH, and PFK), leading to respiratory depression and energy limitation within the cells of *Pseudomonas fragi*. Further studies indicated that it alters intracellular metabolic pathways, with significant changes in amino acid and energy metabolism [53], underscoring its multi-target antimicrobial action.

A comparative analysis of the antimicrobial efficacy of linalool and sodium hypochlorite showed that linalool required about 10-fold higher concentrations to achieve comparable bacterial reduction (Table 2). Our findings align with recent work by Krapež et al. [54], which showed that the MIC of *E. coli* and *S. aureus* exposed to linalool was twice that required for sodium hypochlorite. This consistent observation across studies confirms that while natural antimicrobials such as linalool have promising safety profiles, they generally require significantly higher concentrations (typically from 2 to 10 times higher, depending on bacterial strain) than conventional disinfectants to achieve a comparable effect.

#### 3.2.3. Cell Viability Analysis

**Figure 2:**
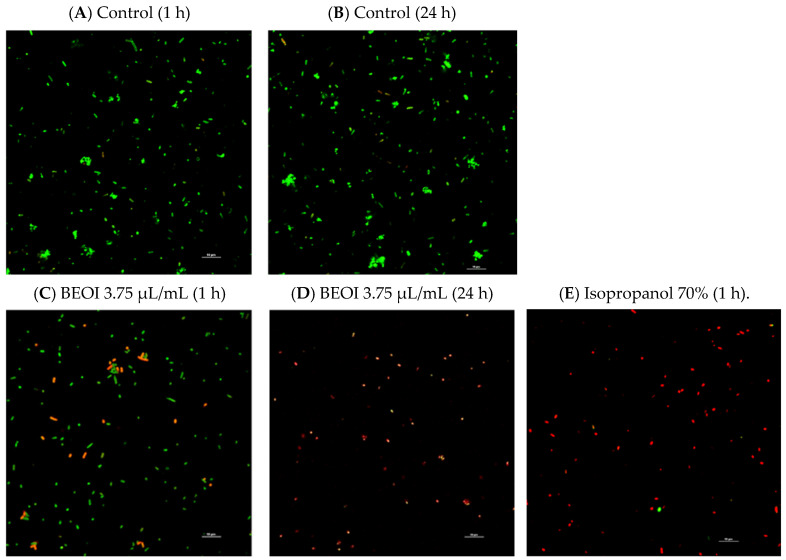
Live and dead cells of *Salmonella enterica* subsp. *salamae* TJC19 visualized by fluorescence microscopy after staining with CFDA (carboxyfuorescein diacetate) and PI (propidium iodide). Control and treated with 3.75% of BEOI after 1 h and 24 h. Live and death as (green) intact bacteria structure; (red) damaged bacteria structure. The experiments were repeated three times, and the representative images are given. Scale bar: 10 μm.

Bacterial cell viability was assessed using dual fluorescent staining with propidium iodide (PI) and carboxyfluorescein diacetate (CFDA) probes. Cells with intact membranes that exhibited green fluorescence (CFDA positive) were classified as viable. While non-viable cells with compromised membranes showed a red fluorescence (PI positive). Overall, the three strains studied showed analogous responses; we report here as an example, the effects on *Salmonella enterica* subsp. *salamae* TJC19. In the control group, the bacteria (Figure 2A,B) effectively bind the CFDA dye, and the visible green fluorescent shade indicates healthy and viable cells after 1 h and 24 h of incubation. The treatment with BEOI led to a conspicuous red fluorescent color that was approximately 27% after 1 h (Figure 2C); this percentage was increased to 96% after 24 h of exposure (Figure 2D), indicating impaired integrity of the cell walls and membranes, ultimately leading to cell death. Figure 2E shows cells exposed to isopropanol (positive control for non-viable cells). Similar behavior was observed in cells treated with linalool. Our findings suggested that BEOI and linalool are effective strategies to combat bacteria with a wide spectrum of drug resistance, likely through rapid membrane disruption.

### 3.3. Biofilm Production

#### 3.3.1. Detection of the Biofilm Morphotype

A biofilm morphotype refers to the distinct physical appearance and structural characteristics of a bacterial biofilm, determined by the composition of its extracellular matrix (EPS) and expression of specific surface components (i.g., curli fimbriae, cellulose) [55]. Depending on curli and/or cellulose expression, the strains can present typical biofilm morphologies based on the color, roughness, and dryness of biofilms formed on Congo Red agar (CR) [23]. CR is an unspecific dye that can bind both polysaccharides (e.g., cellulose) and extracellular proteins (e.g., amyloid fibers) from the biofilm matrix. CR screening helps identify high-risk biofilm-forming strains. Thus, the biofilms produced by *B. oceanisediminis*, *B. thuringensis*, and *S. enterica* subsp. *salamae* were accordingly often categorized into the morphotypes RDAR (red, dry and rough) for strains expressing curli fimbriae and cellulose, BDAR (brown, dry and rough) for strains expressing curli fimbriae, and PDAR (pink, dry and rough) for strains expressing cellulose.

**Figure 3:**
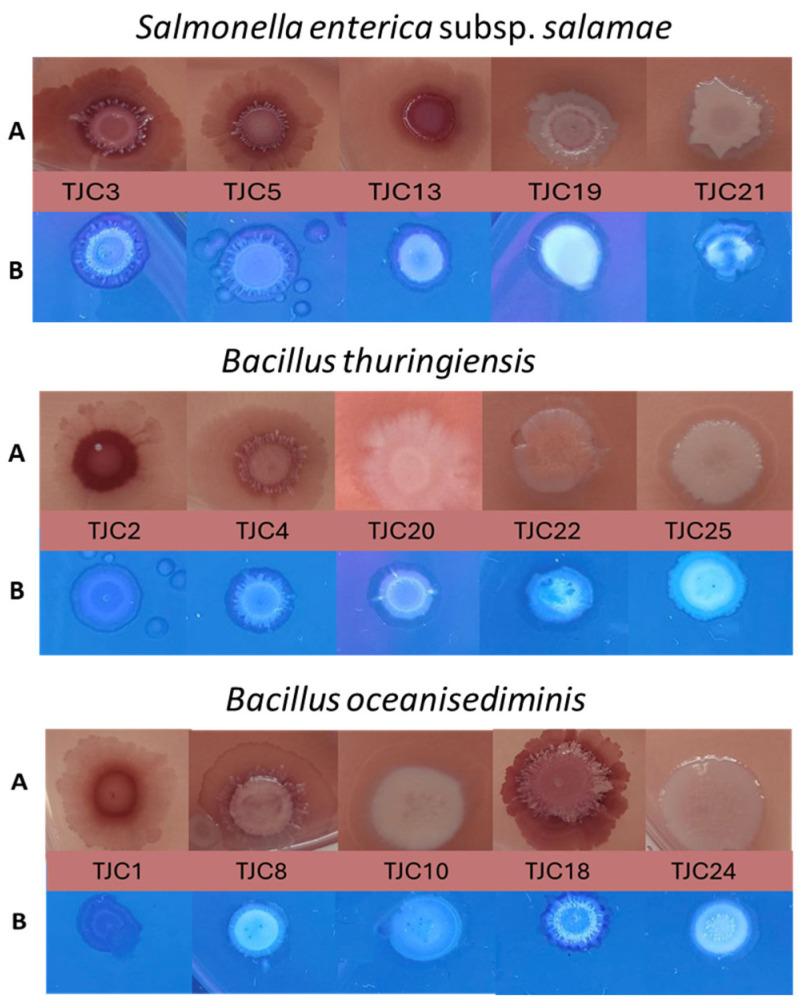
Colony morphotypes on Congo red assays. Photographs of biofilm colonies of *S. enterica* subsp. *salamae, B. oceanisediminis*, *B. thuringensis*. (**A**) Petri-dish CR-agar assays, (**B**) Petri-dish calcofluor agar assays.

Figure 3 shows the images of the colony growth after 8 days at 15 °C, and is representative of at least two independent experiments. As evidence, the biofilm morphology is a strain-specific characteristic. In fact, morphotype RDAR (curli+, cellulose+) was presented only in the strains of *B. oceanisediminis* TJC8, TJC18, *B. thuringensis* TJC4, and *S. enterica* subsp. *salamae* the strains TCJ3, TCJ5 and TCJ13. In addition, the morphotype BDAR (curli+, cellulose−) was displayed only by the strains *B. oceanisediminis* TJC1 and *B. thuringensis* TJC 2, while the morphotype PDAR (curli−, cellulose+) was presented in *S. enterica* subsp. *salamae* TJC19, TJC 21, *B. oceanisediminis* TJC10, TJC 24 and *B. thuringensis* TJC 20, TJC 22, and TJC 25. The cellulose expression pattern of all the strains was also confirmed by the Calcofluor assay.

Castelijn et al. [56] reported that curli fimbriae and cellulose specifically contribute to biofilm production under low-nutrient conditions at ambient temperatures, while other components may play a more significant role in biofilm formation at 37 °C and/or in nutrient-rich conditions. In our study, strains inoculated in FBMS+CR+CBB did not exhibit significant differences in the BDAR and PDAR biofilm morphotypes compared to growth in CR media. However, the RDAR morphotype was less pronounced (Figure S1). Further research is needed to better understand this behavior.

#### 3.3.2. Biofilm Formation Ability of *B. oceanisediminis*, *B. thuringensis*, and *S. enterica* subsp. *salamae* Strains in Two Different Growth Media Inoculated in Polystyrene Microplates

The composition of growth substrates, whether natural or synthetic, significantly affects microbial adhesion and biofilm formation, leading to variations in the structure and function of the microbial community. To improve the relevance of laboratory studies, biofilm formation and/or sessile cell resistance in laboratory experiments with food or media that mimic the conditions that microorganisms are likely to encounter during food processing, thus improving the understanding and prediction of their sessile behavior [57]. In this study, we evaluated the biofilm-forming capacity of all the strains in two different media, TSB and FBMS, which differ substantially in nutritional composition. The main nutrients in TSB include the pancreatic digest of casein (1.7%), papain digest of soybean (0.3%), glucose (0.25%), sodium chloride (0.5%), and dipotassium hydrogen phosphate (0.25%). While FBMS has a complex composition and contains proteins (2.73%), total free amino acids (0.42%), soluble peptides (0.62%), fats (0.54%), and ash (0.89%).

**Figure 4:**
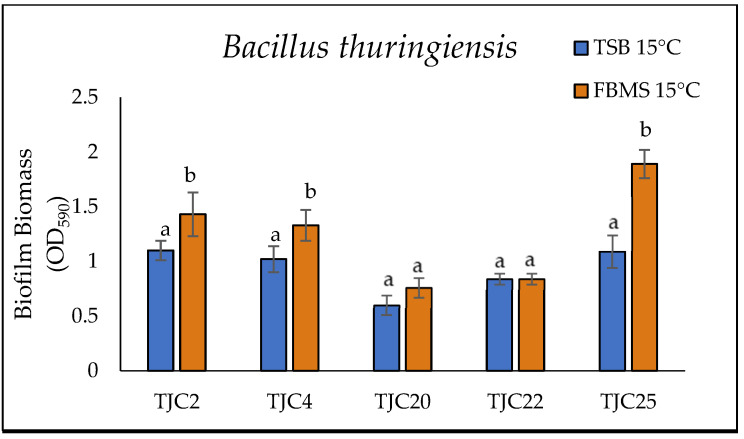

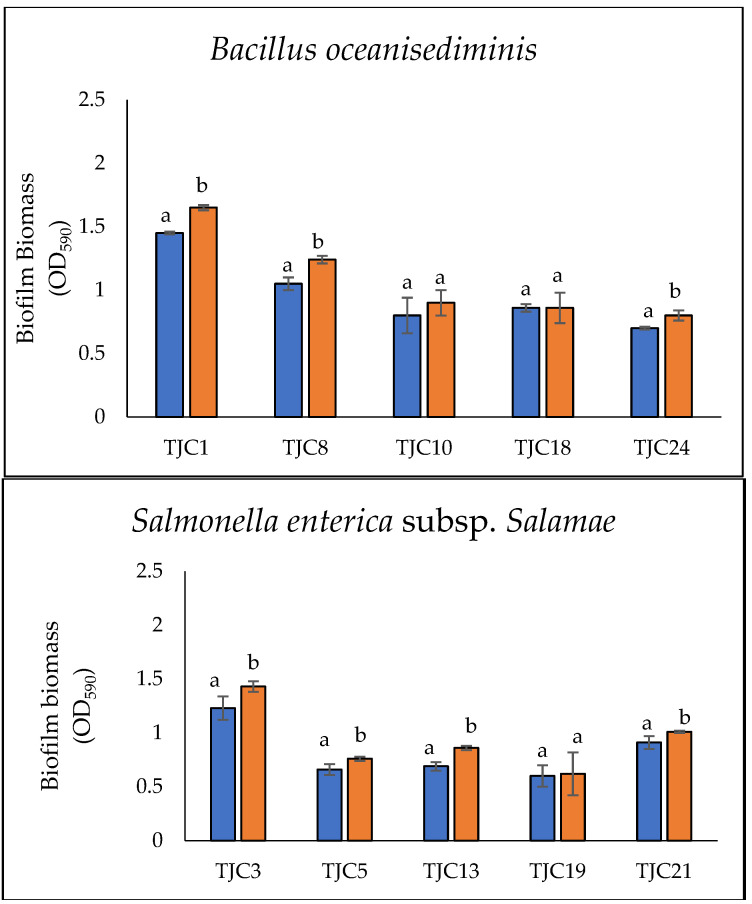
Biofilm biomass (OD_590nm_) of *Bacillus thuringensis*, *Bacillus oceanisediminis*, and *Salmonella enterica* subsp. *salamae* after 144 h at 15 °C of incubation, growth in BHI and FBMS. The results are expressed as the average of five replicates, and the bars represent the standard deviations. The letters indicate a statistically significant difference between the different strains of the same species. The strains were grouped into: OD590 < 0.1, non-producers (NP); OD_590nm_ =0.1–1.0, weak producers (WP); OD_590nm_ =1.1–3.0, moderate producers (MP); and OD_590nm_ > 3.0, strong producers.

Figure 4 illustrates the ability of the strains investigated in this study to develop biofilms on polystyrene surfaces using TSB and FBMS as growth media at a temperature of 15 °C. All strains demonstrated biofilm-forming ability (absorbance at 590 nm ≥ 1 in crystal violet assays), although with different biofilm patterns. Notably, biofilm production was consistently higher in FBMS than in TSB. While a significant proportion (11/15 strains) were classified as weak biofilm producers in TSB, *B. thuringensis* TJC2, and TJC25, *B. oceanisediminis* TJC1, and *S. enterica* subsp. *salamae* TCJ3 exhibited moderate biofilm formation. Conversely, FBMS significantly enhanced biofilm development across most strains, with only 8 of 15 remaining weak producers. Strikingly, *B. thuringensis* TJC25, produced the highest biomass, being classified as a moderate biofilm producer. These observed differences in biofilm formation and phenotypic resistance patterns likely reflect the profound genomic differences between strains as observed previously by Fagerlund et al. [58]. Divergent opinions still exist on the relationship between nutrient availability and the development of biofilms. Some research has found that biofilm formation is decreased under nutrient deprivation, while other studies have suggested the opposite [59]. Additionally, particles of foods may promote the initial bacterial attachment to inert surfaces, promoting biofilm formation [60].

Interestingly, while the RDAR morphotype (cellulose and curli fimbriae production) is often linked to environmental resistance and robust biofilm formation [61], our data indicate that RDAR expression is not strictly necessary for high biofilm production. This observation aligns with previous reports [60], suggesting alternative mechanisms may drive biofilm abundance in certain strains.

On the other hand, the effective biofilm formation observed at 15 °C is particularly relevant, suggesting that these species can form biofilms during food processing and thus facilitate cross-contamination of products. Therefore, all our results support the hypothesis that the ability to form biofilms at 15 °C contributes to the persistence of these species in a fishery production environment. However, further studies with a larger collection of strains would be needed to confirm this relationship.

Our study provides evidence that the choice of culture medium selection is an apparently critical factor for biofilm development for some strains, with food-based medium (FBMS) producing significantly more robust biofilms than conventional TSB (Figure 4). This pronounced medium-dependent discrepancy reveals a critical limitation of traditional laboratory models, which systematically underestimate the biofilm formation potential of food pathogens in real processing environments. The enriched nutrient profile and complex physicochemical properties of FBMS (including fish proteins, lipids, and minerals) appear to (1) stimulate the production of extracellular polymer, (2) enhance bacterial surface adhesion, and (3) promote the formation of architecturally more complex biofilms in some strains. These findings have immediate practical consequences, as the enhanced biofilm formation observed under food-relevant conditions is likely to lead to increased resistance to common disinfectants, possibly explaining the limited efficacy of some disinfectants in industrial environments. We therefore advocate a paradigm shift in biofilm research methodology, where validation in food-like media becomes standard practice to bridge the gap between laboratory results and real-world applications. Future work will focus on how specific food matrix components (e.g., fish protein, calcium) modulate both biofilm architecture and disinfectant penetration using more strains.

#### 3.3.3. Comparison of Biofilm Formation in Polystyrene and Stainless Steel Surfaces

**Figure 5:**
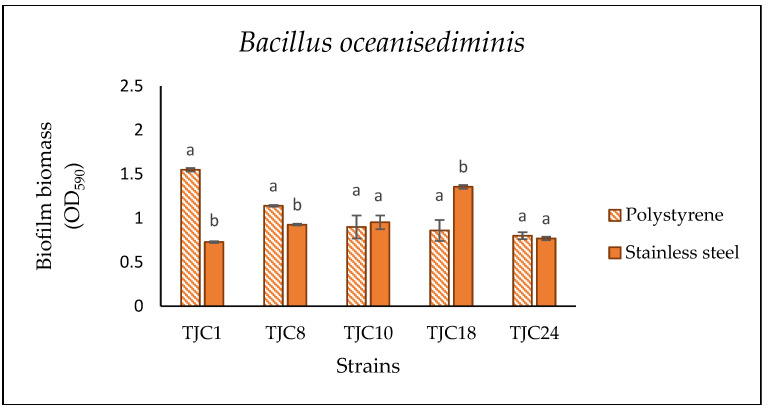

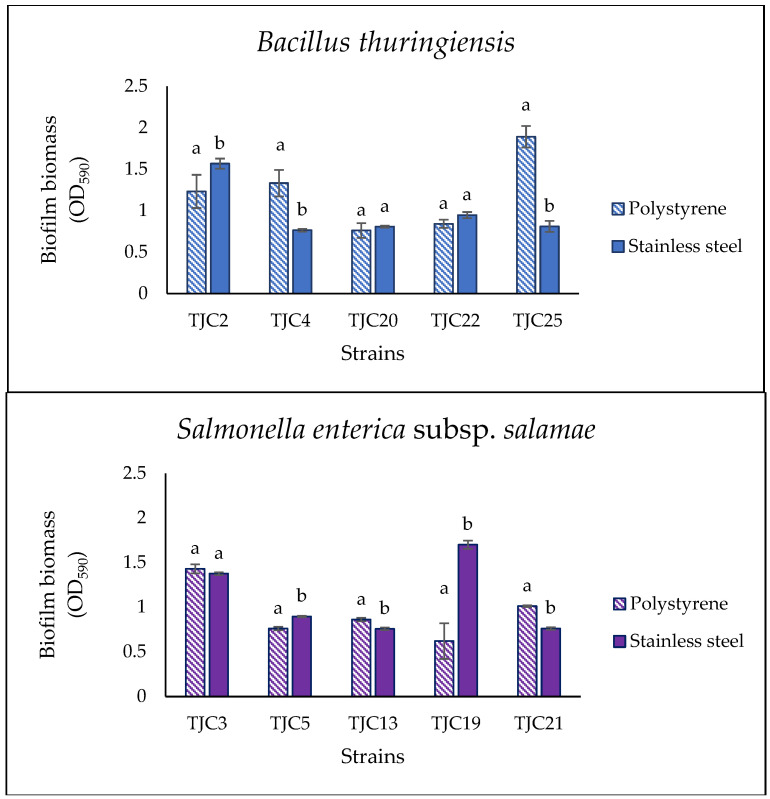
Comparison of the biofilm biomass formed by *Bacillus thuringensis*, *Bacillus oceanisediminis*, and *Salmonella enterica* subsp. *salamae* on polystyrene and stainless steel after 144 h at 15 °C of incubation, growth in FBMS. The results are expressed as the average of five replicates, and the bars represent the standard deviations. The different letters indicate significant differences between biomas formation in the different surface among of the strains.

It is well known that surface properties significantly influence biofilm formation. In the food industry, SS is widely used due to its high mechanical strength, easy cleaning, and compatibility with process equipment. Our results (Figure 5) demonstrated that the SS surface supports the biofilm formation of the different strains with a strain-dependent effect. Notably, B. *oceanisediminis* TJC18, *S. enterica* subsp. *salamae* TJC 19 and *B. thuringensis* TJC2 exhibited higher biofilm biomass on SS compared to PS surfaces, whereas *B. oceanisediminis* TJC1, *B. thuringensis* TJC4, and TJC 25 were the weak producers.

A particularly interesting observation was the stark contrast between *S. enterica* subsp. *salamae* TJC19, an RDAR morphotype (expressing both cellulose and curli fimbriae), which formed a robust biofilm on SS and *B. thuringiensis* TJC4, a SAW morphotype (smooth colonies lacking significant EPS), which demonstrated poor biofilm production. This underlines the critical role of strain-specific traits, including EPS composition, in determining biofilm formation on industrial surfaces. Previous research suggests that bacterial biofilms typically accumulate more biomass on hydrophobic surfaces (e.g., polystyrene) than on hydrophilic ones (e.g., stainless steel) primarily due to differences in surface properties. Hydrophobic interactions play a key role in bacterial adhesion, with a cell surface hydrophobicity strongly correlating with affinity for polymeric materials. Numerous studies support that bacterial attachment is generally stronger on hydrophobic surfaces than on hydrophilic surfaces [61,62,63]. However, our findings align with studies indicating that hydrophobicity is not a decisive factor, as hydrophilic surfaces like stainless steel and glass can also support substantial bacterial adhesion under certain conditions [64,65]. This suggests that additional factors such as surface roughness, conditioning films, or strain-specific traits may modulate biofilm formation, highlighting the complexity of microbial-surface interactions.

#### 3.3.4. Inhibition of Biofilm Formation by Basil Essential Oil and Linalool

This part of the research was focused on evaluating the efficacy of BEO and linalool in inhibiting biofilm formation. We employed BEOI, which showed major antibacterial activity, and FBMS media, which was previously determined to optimally support biofilm production. Thus, to assess BEOI’s anti-biofilm potential, all the studied strains were treated with their respective MIC under standardized conditions.

Table 3 illustrates the impact of BEOI on biofilm formation measured via crystal violet absorbance at 590 nm. The results showed significant differences in the effectiveness of biofilm reduction between the two antimicrobial compounds (BEOI and linalool) and the surfaces studied (polystyrene and stainless steel). Linalool proved particularly powerful on PS, achieving complete (100%) eradication of biofilm on several strains, including *Bacillus oceanisediminis* (TJC1, TJC24), *Bacillus thuringiensis* (TJC20), and *Salmonella enterica* subsp. *salamae* (TJC3, TJC19). In contrast, the reduction in biofilm on SS was generally lower, although remarkable results were observed with BEOI (e.g., *B. thuringiensis* TJC2 with 76.62%) and linalool (e.g., *Salmonella* TJC19 with 77.65%). It is to highlight that the efficacy of both agents varied depending on the strain, with polystyrene proving to be more suitable for treatment than SS. These results emphasized the critical role of the surface material in determining biofilm removal efficiency.

The data suggest that linalool is highly effective on PS, often achieving almost complete biofilm removal. However, on SS, no single antibiofilm agent was consistently better than the other, as efficacy varied significantly depending on the bacterial strain. Therefore, when treating biofilms on SS, the choice of antimicrobial agent (BEOI or linalool) should be adapted to the particular bacterial strain. Further research could investigate surface-specific formulations to improve performance on resistant materials such as SS.

The collective evidence from multiple studies indicates that linalool is effective in inhibiting biofilm formation across a variety of microbial species, including bacteria and fungi. It achieves this by disrupting biofilm architecture, reducing cell motility, and interfering with quorum sensing and gene expression related to biofilm formation. In this context, Wang et al. [66] reported that treatments with 2 µL/mL linalool led to a dose-dependent suppression of biofilm development in *E. coli* that was correlated with a down-regulation of pgaABCD gene expression involved in the production of the exopolysaccharide poly-beta-1,6-N-acetyl-D-glucosamine (PGA), which is crucial for biofilm formation in *E. coli*. In addition, it can disrupt quorum-sensing mechanisms [67,68].

Three-way ANOVA (Table S1) revealed that biofilm reduction was significantly influenced (*p* < 0.0001) by both bacterial strain and surface type across all the tested species. Additionally, the presence of BEO or Linalool significantly reduced the biofilm formation (*p* < 0.0001), though this effect was specific to *B. thuringensis*. Furthermore, the interaction between strain and treatment, as well as the combined effects of strain and surface, significantly impacted the percentage of biofilm reduction. This result underscores the complexity of biofilm dynamics and highlights the need for multifactorial approaches in antibiofilm applications.

#### 3.3.5. Comparison Between the Biofilm Formation Inhibition by Linalool and Sodium Hypochlorite

Since linalool showed more efficacy than BEOI and, in particular, in polystyrene, we decided to compare its efficacy with 0.1% NaClO (the regulatory standard per EPA 2021 and EU 528/2012). From Table 4, it is possible to highlight that 0.1% NaClO achieved 80–95% of biofilm removal efficacy across all strains, significantly outperforming linalool’s 40–60% reduction at equivalent concentrations. While linalool required 2–3× higher doses to match hypochlorite’s effect, it offers critical advantages: no surface corrosion, zero harmful emissions, and sustained activity in organic-rich environments where hypochlorite rapidly degrades. This trade-off between immediate potency (hypochlorite) and sustainable safety (linalool) suggests natural alternatives may better suit applications prioritizing material compatibility and environmental impact, despite needing concentration optimization.

## 4. Conclusions

This study provides novel insights into the inhibitory effects of basil essential oils from Colombia (BEOC) and Italy (BEOI) on the growth and biofilm formation of *Bacillus oceanisediminis*, *Bacillus thuringiensis*, and *Salmonella enterica* subsp. *salamae*. BEOI demonstrated superior efficacy in reducing bacterial cell viability, compared with BEOC, likely due to its high linalool content. The antibacterial activity of BEOI was strain-dependent, with a minimum bactericidal concentration (MBC) ranging between 3.75 and 15 µL/mL. These findings underscore the importance of evaluating multiple strains within the same species to accurately assess the efficacy of essential oils. In addition, our findings clearly demonstrate that the choice of culture medium critically influences biofilm formation, with food-based medium (FBMS) promoting significantly stronger biofilms than standard TSB medium (Figure 3). The stark contrast between media emphasizes that traditional lab media like TSB may underestimate biofilm risks in food processing environments, where nutrient-rich conditions and surface interactions favor persistent biofilm formation. This has critical implications for sanitization protocols, as biofilms formed in food-relevant media may exhibit greater resistance to disinfectants. Future studies should prioritize testing biofilm interventions under conditions that mimic real food matrices to ensure translational relevance.

The results highlight the promising role of BEOI as a natural antimicrobial agent capable of combating multidrug-resistant bacteria and disrupting biofilm formation. Given this efficacy, BEOI is a promising candidate for applications in food processing, healthcare environments, and other settings where controlling drug-resistant pathogens and biofilms is crucial. Further studies will be conducted to investigate the influence of BEOI on bacterial virulence factors, including curli fimbriae and cellulose production, to further elucidate its mechanistic effects.

The observed differences in strain sensitivity to BEO suggest that species-level generalizations should be made cautiously. Strain-specific adaptations or local selection pressures (e.g., those present in Laguna de la Cocha, Pasto, Colombia) may influence these results. Therefore, expanding the range of strains tested in future studies will be essential to better capture natural microbial diversity and optimize treatment protocols.

## Figures and Tables

**Table 1:** Basil essential oil compounds analyzed by the GC-MS method.

	BEOC	BEOI	
Compound	Amount Relative (%)		Kovats Retention Index
**Alcohols**			
1-octen-3-ol	0.28 ± 0.10	N.D.	1003
**Ester**			
Ethyl isovalerate	0.11 ± 0.00	N.D.	853
Iso-bornyl acetate	N.D.	0.92 ± 0.08	1789
**Ether**			
Estragol	10.91 ± 0.34	7.89 ± 0.87	1204
Coumarin	N.D.	0.91 ± 0.01	1890
**Monoterpene oxygenated**			
1,8-cineole (Eucalyptol)	N.D.	12.91 ± 0.52	768
Eucalyptol	24.12 ± 0.97	N.D.	1036
trans-sabinene hydrate	1.25 ± 0.05	N.D.	1072
cis-sabinene hydrate	0.21 ± 0.06	N.D.	1105
Linalool	0.25 ± 0.01	69.86 ± 1.89	1109
Camphor	24.61 ± 1.03	0.77 ± 0.05	1156
4-Terpineol	0.69 ± 0.08	1.02 ± 0.01	1176
α-terpineol	0.46 ± 0.01	0.69 ± 0.01	1184
Nerol	0.11 ± 0.01	N.D.	1234
**Monoterpene not oxygenated**			
α-pinene	1.48 ± 0.10	0.37 ± 0.01	932
terpinolene	N.D.	1.47 ± 0.21	948
Camphene	2.51 ± 0.19	N.D.	947
Sabinene	0.52 ± 0.01	N.D.	973
β-pinene	2.66 ± 0.24	N.D.	976
β-Mircene	0.94 ± 0.01	N.D.	991
2-Carene	0.13 ± 0.01	N.D.	1017
trans-β-Ocimene	0.34 ± 0.01	N.D.	1039
cis-β-Ocimene	2.75 ± 0.39	N.D.	1050
γ-Terpinene	0.27 ± 0.03	N.D.	1060
α-terpinolene	0.41 ± 0.01	N.D.	1089
**Phenylpropene**			
Chavicol	2.43 ± 0.15	N.D.	1282
Eugenol	4.22 ± 0.37	N.D.	1371
**Sesquiterpenes**			
α-Copaene	0.49 ± 0.01	N.D.	1383
β-bourbonene	0.32 ± 0.00	N.D.	1393
β-cubebene	0.25 ± 0.02	N.D.	1396
trans-Cariofilene	2.47 ± 0.04	N.D.	1431
α-bergamotene	1.96 ± 0.02	N.D.	1442
β-Sesquifelandrene	0.23 ± 0.01	N.D.	1448
trans-β-Farnesene	0.17 ± 0.01	N.D.	1458
α-Humulene	0.23 ± 0.01	N.D.	1466
Germacrene D	3.51 ± 0.05	N.D.	1494
β-Bisabolene	2.62 ± 0.04	N.D.	1515
α-Copaene	0.15 ± 0.00	N.D.	1532
α-Bisabolene	5.59 ± 0.36	N.D.	1550
**Oxygenated Sesquiterpenes**			
Viridiflorol	0.27 ± 0.01	N.D.	1600
τ-cadinol	N.D.	5.76 ± 0.35	2213

Values are mean ± standard deviation of two different experiments. N.D.: non detected.

**Table 2:** Minimum inhibitory concentration (MIC; µL/mL) and minimum bactericidal concentration (MBC; µL/mL) of Basil essential oils, linalool, and sodium hypochlorite against bacteria isolated from Rainbow trout (*Oncorhynchus mykiss)* and grown in a fish-based model system.

	BEOC (µL/mL)		BEOI (µL/mL)		Linalool (µL/mL)		NaCLO (µL/mL)	
	MIC	MBC	MIC	MBC	MIC	MBC	MIC	MBC
***B. oceanisediminis***								
TJC1	30	30	3.75	15	0.0125	0.025	0.0023	0.0023
TJC8	30	30	7.5	15	0.0125	0.025	0.0047	0.0047
TJC10	15	30	7.5	7.5	0.0125	0.025	0.0047	0.0047
TJC18	30	30	7.5	7.5	0.0125	0.025	0.0047	0.0047
TJC24	30	30	7.5	7.5	0.00625	0.025	0.0047	0.0047
***B. thuringiensis***								
TJC2	30	30	7.5	7.5	0.0125	0.025	0.0047	0.0047
TJC4	30	30	15	15	0.0125	0.025	0.0047	0.0047
TJC20	30	30	7.5	15	0.00625	0.0125	0.0047	0.0047
TJC22	30	30	15	15	0.0125	0.025	0.0047	0.0047
TJC25	30	30	7.5	15	0.0125	0.025	0.0047	0.0047
***S. enterica* subp. *salamae***								
TJC3	15	30	7.5	15	0.00625	0.0125	0.0047	0.0047
TJC5	30	30	1.87	3.75	0.00625	0.025	0.0047	0.0047
TJC13	30	30	3.75	7.5	0.0125	0.025	0.0047	0.0047
TJC19	30	30	3.75	7.5	0.00625	0.0125	0.0047	0.0047
TJC21	30	30	3.75	7.5	0.0125	0.025	0.0047	0.0047

**Table 3:** Effect of *O. basilicum* oil from Italy (BOEI) and linalool on biofilm biomass reduction in Bacillus *thuringiensis* (a), *Bacillus oceanisediminis* (b) *Salmonella enterica* subsp. *salamae* (c) on polystyrene and stainless steel surfaces after 144 h at 15 °C of incubation.

*B. oceanisediminis*	Treatment	Stainless Steel	Polystyrene
TJC1	BEOI *	35.18 ± 2.90 B jk	69.33 ± 0.97 A cdef
TJC8		48.09 ± 0.96 B hijk	78.19 ± 9.95 A bcde
TJC10		48.85 ± 0.50 B ghijk	84.92 ± 0.04 A abc
TJC18		67.46 ± 3.51 B cdefg	81.02 ± 2.82 A abcd
TJC24		33.98 ± 1.33 B k	64.95 ± 8.11 A defgh
TJC1	LIN *	48.07 ± 0.98 B hijk	100 ± 0.83 A a
TJC8		40.77 ± 1.40 B jk	53.27 ± 8.09 A fghij
TJC10		61.74 ± 1.38 B	79.26 ±8.62 A bcde
TJC18		39.99 ± 3.29 B jk	45.61 ± 7.44 A ijk
TJC24		43.79 ± 0.99 B ijk	92.89 ± 6.15 A ab
***B. thuringiensis***	**Treatment**	**Stainless steel**	**Polystyrene**
TJC2	BEOI *	76.62 ± 3.25 B bc	85.12 ± 3 A ab
TJC4		4.06 ± 1.85 B i	77.61 ± 1.43 A bc
TJC20		3.88 ± 2.29 B i	79.37 ± 1.29 A bc
TJC22		45.14 ± 1.17 B gh	71.69 ± 6.18 A bcd
TJC25		46.60 ± 1.75 B fgh	68.88 ± 4.09 A bcde
TJC2	LIN $	54.66 ± 8.08 B defg	80.14 ± 3.11 A bc
TJC4		50.68 ± 0.80 B efgh	80.70 ± 8.83 A bc
TJC20		64.18 ± 2.78 B cdef	100 ± 7.88 A a
TJC22		50.30 ± 0.90 B efgh	75.75 ± 6.41 A bc
TJC25		34.75 ± 1.11 B h	77.03 ± 8.63 A bc
***S. enterica* subsp. *salamae***	**Treatment**	**Stainless steel**	**Polystyrene**
TJC3	BEOI *	47.27 ± 2.10 B cde	78.47 ± 7.15 A ab
TJC5		39.88 ± 1.20 B de	89.33 ± 16.39 A a
TJC13		30.73 ± 0.45 B de	82.54 ± 11.82 A ab
TJC19		64.99 ± 0.28 B bc	89.7 ± 2.96 A a
TJC21		47.51 ± 2.33 B cd	89.75 ± 0.24 A a
TJC3	LIN *	62.90 ± 3.19 B bc	100 ± 3.48 A a
TJC5		37.28 ± 3.60 B de	78.38 ± 5.39 A ab
TJC13		24.61 ± 0.91 B e	84.09 ± 3.21 A ab
TJC19		77.65 ± 0.57 B ab	98.08 ± 5.46 A a
TJC21		33.57 ± 1.20 B de	92.26 ± 6.56 A a

Results are the media and standard deviation of three repetitions. Different upper-case letters represent statistically significant differences between materials. Different lower-case letters represent statistically significant differences among strains of the same species related to treatments and materials. Different symbols represent statistical differences among treatments (Tukey HSD test; *p* < 0.0001).

**Table 4:** Comparison between the biofilm formation inhibition by linalool and NaClO.

Biofilm Formation Inhibition (%)		
	Linalool	NaClO
*B. oceanisediminis*		
TJC1	100 ± 0.83 a	99.51 ± 0 a
TJC8	53.27 ± 8.09 b	95.25 ± 1.91 a
TJC10	79.26 ±8.62 a	97.64 ± 1.66 a
TJC18	45.61 ± 7.44 b	97.00 ± 1.53 a
TJC24	92.89 ± 6.15 a	99.74 ± 0.35 a
*B. thuringiensis*		
TJC2	80.14 ± 3.11 b	95.35 ± 0.43 a
TJC4	80.70 ± 8.83 a	97.13 ± 1.35 a
TJC20	100 ± 7.88 a	94.56 ± 3.49 a
TJC22	75.75 ± 6.41 a	93.70 ± 2.42 a
TJC25	77.03 ± 8.63 a	96.76 ± 0.24 a
*S. enterica* subsp. *salamae*		
TJC3	100 ± 3.48 a	98.85 ± 0.65 a
TJC5	78.38 ± 5.39 b	98.43 ± 0 a
TJC13	84.09 ± 3.21 b	97.09 ± 0.82 a
TJC19	98.08 ± 5.46 a	98.20 ± 1.26 a
TJC21	92.26 ± 6.56 a	96.15 ± 4.35 a

Different letterrs mean significant differences between linalool and NaClO treatment.

## Data Availability

The original contributions presented in this study are included in the article/Supplementary Materials. Further inquiries can be directed to the corresponding authors.

## Supplementary Materials

The following supporting information can be downloaded at https://www.mdpi.com/article/10.3390/foods14101830/s1, Figure S1: Identification of the bacterial strain isolates from fresh rainbow trout (*Oncorhynchus mykiss*) from Laguna de la Cocha (Pasto, Colombia) determined by 16S rRNA encoding gene sequence analysis. Table S1. Three-way ANOVA for studying the effect of different treatments, materials on the different strains of the same species.

## Abbreviations

The following abbreviations are used in this manuscript:

BEOC	Essential oils of *Ocimum basilicum* L., from Colombia
BEOI	Essential oils of *Ocimum basilicum* L., from Italy
FBMS	Fish-based model system
MIC	Minimum Inhibitory Concentration
MBC	Minimum bactericidal concentration
PI	Propidium iodide
CFDA	Carboxyfluorescein diacetate
BHI	Brain Heart Infusion Broth
TSB	Tryptic soy broth
TTC	2,3,5-triphenyltetrazolium chloride
CLSM	Confocal laser scanning microscopy
E	Erythromicin
AML	Amoxicillin
CXM	Cefuroxime
TE	Tetracycline
TEC	Teicoplanin
C	Chloramphenicol
K	Kanamicyn
DXT	Doxycyclin
OX	Oxacillin
CN	Gentamicin
LNZ	Linezolid
ENR	Enrofloxacin
Na	Nalidixic acid
CD	Clindamycin
RD	Rifampicin
AK	Amikacin
KF	Cephalothin
CIP	Ciprofloxacin
SXT	Sulphamethoxalone
B	Bacitracin
UB	Flumequine
SP	Spiramycin
OL	Oleandomycin
FD	Fusidic acid

## References

[1] Xiong H., Zhou X., Cao Z., Xu A., Dong W., Jiang M. (2024). Microbial biofilms as a platform for diverse biocatalytic applications. Bioresour. Technol..

[2] Carrascosa C., Raheem D., Ramos F., Saraiva A., Raposo A. (2021). Microbial Biofilms in the Food Industry—A Comprehensive Review. Int. J. Environ. Res. Public Health.

[3] Maggio F., Rossi C., Chaves-López C., Serio A., Valbonetti L., Pomilio F., Chiavaroli A.P., Paparella A. (2021). Interactions between *L. monocytogenes* and *P. fluorescens* in Dual-Species Biofilms under Simulated Dairy Processing Conditions. Foods.

[4] Rossi C., Chaves-López C., Serio A., Casaccia M., Maggio F., Paparella A. (2022). Effectiveness and mechanisms of essential oils for biofilm control on food-contact surfaces: An updated review. Crit. Rev. Food Sci. Nutr..

[5] Tadielo L.E., dos Santos E.A.R., Possebon F.S., Schmiedt J.A., Juliano L.C.B., Cerqueira-Cézar C.K., de Oliveira J.P., Sampaio A.N.d.C.E., Melo P.R.L., Caron E.F.F. (2023). Characterization of microbial ecology, *Listeria monocytogenes*, and *Salmonella* sp. on equipment and utensil surfaces in Brazilian poultry, pork, and dairy industries. Food Res. Int..

[6] Sheng L., Wang L. (2021). The microbial safety of fish and fish products: Recent advances in understanding its significance, contamination sources, and control strategies. Compr. Rev. Food Sci. Food Saf..

[7] Liu D., Huang Q., Gu W., Zeng X.-A. (2022). A review of bacterial biofilm control by physical strategies. Crit. Rev. Food Sci. Nutr..

[8] Fernandes S., Gomes I.B., Simões M., Simões L.C. (2024). Novel chemical-based approaches for biofilm cleaning and disinfection. Curr. Opin. Food Sci..

[9] Galié S., García-Gutiérrez C., Miguélez E.M., Villar C.J., Lombó F. (2018). Biofilms in the Food Industry: Health Aspects and Control Methods. Front. Microbiol..

[10] Verma N., Agarwal V. (2022). A Review on Current Strategies for Biofilm Control in Food Industry BT. Proceedings of the Conference BioSangam 2022: Emerging Trends in Biotechnology (BIOSANGAM 2022).

[11] Elafify M., Liao X., Feng J., Ahn J., Ding T. (2024). Biofilm formation in food industries: Challenges and control strategies for food safety. Food Res. Int..

[12] González-Rivas F., Ripolles-Avila C., Fontecha-Umaña F., Ríos-Castillo A.G., Rodríguez-Jerez J.J. (2018). Biofilms in the Spotlight: Detection, Quantification, and Removal Methods. Compr. Rev. Food Sci. Food Saf..

[13] Özdemir F., Arslan S. (2018). Biofilm Production and Antimicrobial Susceptibility Profiles of *Bacillus* spp. from Meats. Sak. Univ. J. Sci..

[14] Park K.M., Kim A.Y., Kim H.J., Cho Y.S., Koo M. (2022). Prevalence and characterization of toxigenic *Bacillus cereus* group isolated from low-moisture food products. Food Sci. Biotechnol..

[15] CLSI (2018). Performance Standards for Antimicrobial Disk Susceptibility Tests.

[16] Chaves-López C., Serio A., Gianotti A., Sacchetti G., Ndagijimana M., Ciccarone C., Stellarini A., Corsetti A., Paparella A. (2015). Diversity of food-borne *Bacillus* volatile compounds and influence on fungal growth. J. Appl. Microbiol..

[17] Pilevar Z., Hosseini H., Abdollahzadeh E., Shojaee-Aliabadi S., Tajedin E., Yousefi M., Bahrami A., Khosroshahi N.K. (2020). Effect of *Zataria multiflora* Boiss. Essential oil, time, and temperature on the expression of *Listeria monocytogenes* virulence genes in broth and minced rainbow trout. Food Control.

[18] Moore S., Stein W.H. (1948). Photometric ninhydrin method for use in the chromatography of amino acids. J. Biol. Chem..

[19] Cheng Y.-S., Zheng Y., VanderGheynst J.S. (2011). Rapid Quantitative Analysis of Lipids Using a Colorimetric Method in a Microplate Format. Lipids.

[20] CLSI (2016). Performance Standards for Antimicrobial Susceptibility Testing. CLSI 448 Supplement M100S.

[21] Molina-Hernandez J.B., Aceto A., Bucciarelli T., Paludi D., Valbonetti L., Zilli K., Scotti L., Chaves-López C. (2021). The membrane depolarization and increase intracellular calcium level produced by silver nanoclusters are responsible for bacterial death. Sci. Rep..

[22] Lee W., Kim K.-J., Lee D.G. (2014). A novel mechanism for the antibacterial effect of silver nanoparticles on *Escherichia coli*. BioMetals.

[23] Choong F.X., Huzell S., Rosenberg M., Eckert J.A., Nagaraj M., Zhang T., Melican K., Otzen D.E., Richter-Dahlfors A. (2021). A semi high-throughput method for real-time monitoring of curli producing *Salmonella* biofilms on air-solid interfaces. Biofilm.

[24] Rossi C., Serio A., Chaves-López C., Anniballi F., Auricchio B., Goffredo E., Cenci-Goga B.T., Lista F., Fillo S., Paparella A. (2018). Biofilm formation, pigment production and motility in *Pseudomonas* spp. isolated from the dairy industry. Food Control.

[25] Rossi C., Maggio F., Chaves-López C., Valbonetti L., Berrettoni M., Paparella A., Serio A. (2022). Effectiveness of selected essential oils and one hydrolate to prevent and remove *Listeria monocytogenes* biofilms on polystyrene and stainless steel food-contact surfaces. J. Appl. Microbiol..

[26] Lamas A., Miranda J.M., Regal P., Vázquez B., Franco C.M., Cepeda A. (2018). A comprehensive review of non-enterica subspecies of *Salmonella enterica*. Microbiol. Res..

[27] Baniga Z., Mdegela R.H., Lisa B., Kusiluka L.J.M., Dalsgaard A. (2019). Prevalence and characterisation of Salmonella Waycross and *Salmonella enterica* subsp. salamae in Nile perch (*Lates niloticus*) of Lake Victoria, Tanzania. Food Control.

[28] EFSA European Food Safety Authority. *Salmonella*. https://www.efsa.europa.eu/en/topics/topic/salmonella.

[29] Kyule D.N., Maingi J.M., Njeru E.M., Nyamache A.K. (2022). Molecular Characterization and Diversity of Bacteria Isolated from Fish and Fish Products Retailed in Kenyan Markets. Int. J. Food Sci..

[30] Ren W., Li P., Wang X., Che Y., Long H., Zhang X., Cai X., Huang A., Zeng Y., Xie Z. (2022). Cross-habitat distribution pattern of *Bacillus* communities and their capacities of producing industrial hydrolytic enzymes in Paracel Islands: Habitat-dependent differential contributions of the environment. J. Environ. Manag..

[31] Dhayalan A., Velramar B., Govindasamy B., Ramalingam K.R., Dilipkumar A., Pachiappan P. (2022). Isolation of a bacterial strain from the gut of the fish, *Systomus sarana*, identification of the isolated strain, optimized production of its protease, the enzyme purification, and partial structural characterization. J. Genet. Eng. Biotechnol..

[32] Bravo A., Gill S.S., Soberón M. (2007). Mode of action of *Bacillus thuringiensis* Cry and Cyt toxins and their potential for insect control. Toxicon.

[33] Nnadozie C.F., Odume O.N. (2019). Freshwater environments as reservoirs of antibiotic resistant bacteria and their role in the dissemination of antibiotic resistance genes. Environ. Pollut..

[34] Czekalski N., Sigdel R., Birtel J., Matthews B., Bürgmann H. (2015). Does human activity impact the natural antibiotic resistance background? Abundance of antibiotic resistance genes in 21 Swiss lakes. Environ. Int..

[35] Carovic-Stanko K., Petek M., Grdisa M., Pintar J., Bedekovic D., Herak Custic M., Satovic Z. (2016). Medicinal plants of the family Lamiaceae as functional foods—A review. Czech J. Food Sci..

[36] Paulus D., Valmorbida R., Ferreira S.B., Zorzzi1 I.C., Nava G.A. (2016). Biomassa e composição do óleo essencial de manjericão cultivado sob malhas fotoconversoras e colhido em diferentes épocas. Hortic. Bras..

[37] Tursun A.O. (2022). Impact of soil types on chemical composition of essential oil of purple basil. Saudi J. Biol. Sci..

[38] Daneshian A., Gurbuz B., Cosge B., Ipek A. (2019). Chemical Components of Essential Oils from Basil (*Ocimum basilicum* L.) Grown at Different Nitrogen Levels. Int. J. Nat. Eng. Sci..

[39] Cheliku N., Cvetkovikj Karanfilova I., Stefkov G., Karapandzova M., Bardhi N., Qjazimi B., Kulevanova S. (2015). Essential oil composition of five Basil cultivars (*Ocimum basilicum*) from Albania. Maced. Pharm. Bull..

[40] Amor G., Sabbah M., Caputo L., Idbella M., De Feo V., Porta R., Fechtali T., Mauriello G. (2021). Basil Essential Oil: Composition, Antimicrobial Properties, and Microencapsulation to Produce Active Chitosan Films for Food Packaging. Foods.

[41] Massoud H.Y., Farag A.A., El-Gamal S.M.A., El-Nabawy S.A.E. (2024). Effect of Different Drip Irrigation Rates and Potassium Silicate Spray on Growth and Essential Oil Production of French Basil. J. Plant Prod..

[42] Viña A., Murillo E. (2003). Essential Oil Composition from Twelve Varieties of Basil (*Ocimum* spp) Grown in Colombia. J. Braz. Chem. Soc..

[43] Snoussi M., Dehmani A., Noumi E., Flamini G., Papetti A. (2016). Chemical composition and antibiofilm activity of *Petroselinum crispum* and *Ocimum basilicum* essential oils against *Vibrio* spp. strains. Microb. Pathog..

[44] Rajaraman S.K., Jainu M., Dhakshinamoorthy G. (2016). *Ocimum basilicum* L. essential oil coated biomaterial surfaces prevent bacterial adhesion and biofilm growth. Asian J. Pharm. Clin. Res..

[45] Rattanachaikunsopon P., Phumkhachorn P. (2010). Antimicrobial Activity of Basil (*Ocimum basilicum*) Oil against *Salmonella enteritidis* in Vitro and in Food. Biosci. Biotechnol. Biochem..

[46] Radaelli M., Silva B.P.d., Weidlich L., Hoehne L., Flach A., Costa L.A.M.A.d., Ethur E.M. (2016). Antimicrobial activities of six essential oils commonly used as condiments in Brazil against *Clostridium perfringens*. Braz. J. Microbiol..

[47] Lv F., Liang H., Yuan Q., Li C. (2011). In vitro antimicrobial effects and mechanism of action of selected plant essential oil combinations against four food-related microorganisms. Food Res. Int..

[48] Hussain A.I., Anwar F., Hussain Sherazi S.T., Przybylski R. (2008). Chemical composition, antioxidant and antimicrobial activities of basil (*Ocimum basilicum*) essential oils depends on seasonal variations. Food Chem..

[49] Bassolé I.H.N., Lamien-Meda A., Bayala B., Tirogo S., Franz C., Novak J., Nebié R.C., Dicko M.H. (2010). Composition and Antimicrobial Activities of *Lippia multiflora* Moldenke, *Mentha x piperita* L. and *Ocimum basilicum* L. Essential Oils and Their Major Monoterpene Alcohols Alone and in Combination. Molecules.

[50] Guo F., Chen Q., Liang Q., Zhang M., Chen W., Chen H., Yun Y., Zhong Q., Chen W. (2021). Antimicrobial Activity and Proposed Action Mechanism of Linalool Against *Pseudomonas fluorescens*. Front. Microbiol..

[51] Bua A., Usai D., Donadu M.G., Delgado Ospina J., Paparella A., Chaves-Lopez C., Serio A., Rossi C., Zanetti S., Molicotti P. (2018). Antimicrobial activity of *Austroeupatorium inulaefolium* (H.B.K.) against intracellular and extracellular organisms. Nat. Prod. Res..

[52] Silva C.G., Yudice E.D.C., Campini P.A.L., Rosa D.S. (2021). The performance evaluation of Eugenol and Linalool microencapsulated by PLA on their activities against pathogenic bacteria. Mater. Today Chem..

[53] Li Y., Ren F., Chen D., Chen H., Chen W. (2022). Antibacterial Mechanism of Linalool against *Pseudomonas fragi*: A Transcriptomic Study. Foods.

[54] Krapež P., Lunder M., Oder M., Fink R. (2024). Evaluation of the In Vitro Disinfection Potential of the Phytochemicals Linalool and Citronellal Against Biofilms Formed by *Escherichia coli* and *Staphylococcus aureus*. Processes.

[55] Hobley L., Harkins C., MacPhee C.E., Stanley-Wall N.R. (2015). Giving structure to the biofilm matrix: An overview of individual strategies and emerging common themes. FEMS Microbiol. Rev..

[56] Castelijn G.A.A., van der Veen S., Zwietering M.H., Moezelaar R., Abee T. (2012). Diversity in biofilm formation and production of curli fimbriae and cellulose of *Salmonella typhimurium* strains of different origin in high and low nutrient medium. Biofouling.

[57] Papaioannou E., Giaouris E.D., Berillis P., Boziaris I.S. (2018). Dynamics of biofilm formation by *Listeria monocytogenes* on stainless steel under mono-species and mixed-culture simulated fish processing conditions and chemical disinfection challenges. Int. J. Food Microbiol..

[58] Fagerlund A., Langsrud S., Heir E., Mikkelsen M.I., Møretrø T. (2016). Biofilm Matrix Composition Affects the Susceptibility of Food Associated *Staphylococci* to Cleaning and Disinfection Agents. Front. Microbiol..

[59] Liu Y., Zhu H., Dou X., Jia K., Panagou E.Z., Zhang H., Xu A., Dong Q. (2024). The influence of nutrients on biofilm formation of an ST87 strain of *Listeria monocytogenes*. LWT.

[60] Paz-Méndez A.M., Lamas A., Vázquez B., Miranda J.M., Cepeda A., Franco C.M. (2017). Effect of Food Residues in Biofilm Formation on Stainless Steel and Polystyrene Surfaces by *Salmonella enterica* Strains Isolated from Poultry Houses. Foods.

[61] Steenackers H., Hermans K., Vanderleyden J., De Keersmaecker S.C.J. (2012). *Salmonella* biofilms: An overview on occurrence, structure, regulation and eradication. Food Res. Int..

[62] Dantas T., Padrão J., da Silva M.R., Pinto P., Madeira S., Vaz P., Zille A., Silva F. (2021). Bacteria co-culture adhesion on different texturized zirconia surfaces. J. Mech. Behav. Biomed. Mater..

[63] Obe T., Richards A.K., Shariat N.W. (2022). Differences in biofilm formation of *Salmonella serovars* on two surfaces under two temperature conditions. J. Appl. Microbiol..

[64] Nguyen P.Q., Botyanszki Z., Tay P.K.R., Joshi N.S. (2014). Programmable biofilm-based materials from engineered curli nanofibres. Nat. Commun..

[65] Monteagudo-Mera A., Rastall R.A., Gibson G.R., Charalampopoulos D., Chatzifragkou A. (2019). Adhesion mechanisms mediated by probiotics and prebiotics and their potential impact on human health. Appl. Microbiol. Biotechnol..

[66] Wang L., Wang J., Zhang K., Zhang J., Cui D., Wang J., Ji P., Wei Y., Li J. (2025). Linalool as a Potential Agent for Inhibiting Escherichia coli Biofilm Formation and Exopolysaccharide Production. BMC Vet. Res..

[67] Shen Y., Gao S., Fan Q., Zuo J., Wang Y., Yi L., Wang Y. (2023). New antibacterial targets: Regulation of quorum sensing and secretory systems in zoonotic bacteria. Microbiol. Res..

[68] Praseetha S., Sukumaran S.T., Dan M., Augustus A.R., Pandian S.K., Sugathan S. (2023). The Anti-Biofilm Potential of Linalool, a Major Compound from *Hedychium larsenii*, against *Streptococcus pyogenes* and Its Toxicity Assessment in *Danio rerio*. Antibiotics.

