# Supplementary material for: Antibiofilm Power of Basil Essential Oil Against Fish-Originated Multidrug-Resistant Salmonella and Bacillus spp.: Targeting Biofilms on Food Contact Surfaces
Source: Foods. 2025 May 21;14(10):1830. doi: 10.3390/foods14101830 (PMC12110890; doi:10.3390/foods14101830)
Supplement: Supplementary file 1 [file foods-14-01830-s001.zip › foods-3594544-supplementary.pdf]

# Antibiofilm Power of Basil Essential Oil Against Fish-Originated Multidrug-Resistant *Salmonella* and *Bacillus* spp.: Targeting Biofilms on Food Contact Surfaces

**Valentina Pavone [1,†], Francisco Argote-Vega [2], Waleed Butt [1,†], Junior Bernardo Molina-Hernandez [1,3,*], Domenico Paludi [4], Johannes Delgado-Ospina [5], Luca Valbonetti [1], José Ángel Pérez-Álvarez [2] and Clemencia Chaves-López [1,*]**

1 Faculty of Bioscience and Technology for Food, Agriculture and Environment, University of Teramo, Via R. Balzarini 1, 64100 Teramo, Italy; vpavone@unite.it (V.P.); wbutt@unite.it (W.B.); lvalbonetti@unite.it (L.V.)
2 IPOA Research Group, Centro de investigación e Innovación Agroalimentaria y Agroambiental de la UMH (CIAGRO), Miguel Hernández University, Orihuela 03202, Alicante, Spain; argote_71@hotmail.com (F.A.-V.); ja.perez@goumh.umh.es (J.Á.P.-Á.)
3 Department of Agricultural and Food Sciences, University of Bologna, 47521 Cesena, Italy
4 Faculty of Veterinary Medicine, University of Teramo, 64100 Teramo, Italy; dpaludi@unite.it
5 Grupo de Investigación Biotecnología, Facultad de Ingeniería, Universidad de San Buenaventura Cali, Carrera 122 # 6-65, 76001 Cali, Colombia; jdelgado1@usbcali.edu.co
* Correspondence: junior.molina@unibo.it (J.B.M.-H.); cchaveslopez@unite.it (C.C.-L.)
† These authors contributed equally to this work.

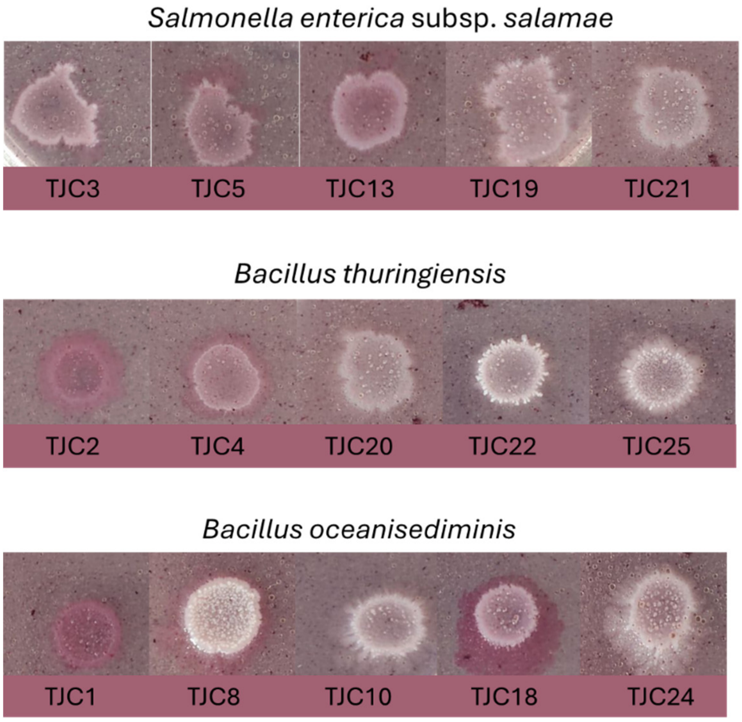


**Figure S1.** Biofilm colony morphotypes of *S. enterica* subsp. *salamae*, *B. oceanisediminis*, *B. thuringensis*.on fish model media supplemented with Congo red and Coomassie brilliant blue G-250.

**Table S1.** Three-way ANOVA for studying the effect of different treatments, materials on the different strains of the same species.

***Salmonella enterica* subsp. *salamae***

| Source | DF | Sum of squares | Mean squares | F | Pr > F | p-values signification codes |
|---|---|---|---|---|---|---|
| strain | 4.000 | 3491.278 | 872.819 | 27.680 | **<0.0001** | *** |
| treatment (BEO-LIN) | 1.000 | 82.133 | 82.133 | 2.605 | 0.122 | ° |
| surface (Polystyrene-Stainless steel) | 1.000 | 17325.472 | 17325.472 | 549.454 | **<0.0001** | *** |
| strain*treatment | 4.000 | 997.069 | 249.267 | 7.905 | **0.001** | *** |
| strain*surface | 4.000 | 1414.411 | 353.603 | 11.214 | **<0.0001** | *** |
| treatment*surface | 1.000 | 30.272 | 30.272 | 0.960 | 0.339 | ° |
| strain*treatment*surface | 4.000 | 195.930 | 48.983 | 1.553 | 0.225 | ° |

*Signification codes: 0 < *** < 0.001 < ** < 0.01 < * < 0.05 < . < 0.1 < ° < 1*

***B. thuringienisis***

| Source | DF | Sum of squares | Mean squares | F | Pr > F | p-values signification codes |
|---|---|---|---|---|---|---|
| strain | 4.000 | 2000.761 | 500.190 | 23.122 | **<0.0001** | *** |
| treatment (BEO-LIN) | 1.000 | 1192.661 | 1192.661 | 55.132 | **<0.0001** | *** |
| surface (Polystyrene-Stainless steel) | 1.000 | 13355.300 | 13355.300 | 617.359 | **<0.0001** | *** |
| strain*treatment | 4.000 | 3729.027 | 932.257 | 43.094 | **<0.0001** | *** |
| strain*surface | 4.000 | 2218.106 | 554.527 | 25.633 | **<0.0001** | *** |
| treatment*surface | 1.000 | 224.062 | 224.062 | 10.357 | **0.004** | ** |
| strain*treatment*surface | 4.000 | 1855.216 | 463.804 | 21.440 | **<0.0001** | *** |

*Signification codes: 0 < *** < 0.001 < ** < 0.01 < * < 0.05 < . < 0.1 < ° < 1*

***B. oceanisediminis***

| Source | DF | Sum of squares | Mean squares | F | Pr > F | p-values signification codes |
|---|---|---|---|---|---|---|
| strain | 4.000 | 874.617 | 218.654 | 9.763 | **0.000** | *** |
| treatment (BEO - LIN) | 1.000 | 4.327 | 4.327 | 0.193 | 0.665 | ° |
| surface (Polystyrene - Stainless steel) | 1.000 | 7926.608 | 7926.608 | 353.936 | **<0.0001** | *** |
| strain*treatment | 4.000 | 4179.315 | 1044.829 | 46.653 | **<0.0001** | *** |
| strain*surface | 4.000 | 1512.328 | 378.082 | 16.882 | **<0.0001** | *** |
| treatment*surface | 1.000 | 6.687 | 6.687 | 0.299 | 0.591 | ° |
| strain*treatment*surface | 4.000 | 673.995 | 168.499 | 7.524 | **0.001** | *** |

*Signification codes: 0 < *** < 0.001 < ** < 0.01 < * < 0.05 < . < 0.1 < ° < 1*
